# Fate of Biodegradable Engineered Nanoparticles Used in Veterinary Medicine as Delivery Systems from a One Health Perspective

**DOI:** 10.3390/molecules26030523

**Published:** 2021-01-20

**Authors:** Constantin Cerbu, Melanie Kah, Jason C. White, Carlos E. Astete, Cristina M. Sabliov

**Affiliations:** 1 Department of Infectious Diseases, Faculty of Veterinary Medicine, University of Agricultural Sciences and Veterinary Medicine Cluj-Napoca, 400372 Cluj-Napoca, Romania; 2 School of Environment, The University of Auckland, Auckland 1010, New Zealand; melanie.kah@auckland.ac.nz; 3 Connecticut Agricultural Experiment Station, New Haven, CT 06511, USA; Jason.White@ct.gov; 4 Biological and Agricultural Engineering Department, Louisiana State University and LSU Agricultural Center, Baton Rouge, LA 70803, USA; castete@agcenter.lsu.edu

**Keywords:** nanoparticles, veterinary medicine, one health, antibiotics, hormones, vaccines

## Abstract

The field of veterinary medicine needs new solutions to address the current challenges of antibiotic resistance and the need for increased animal production. In response, a multitude of delivery systems have been developed in the last 20 years in the form of engineered nanoparticles (ENPs), a subclass of which are polymeric, biodegradable ENPs, that are biocompatible and biodegradable (pbENPs). These platforms have been developed to deliver cargo, such as antibiotics, vaccines, and hormones, and in general, have been shown to be beneficial in many regards, particularly when comparing the efficacy of the delivered drugs to that of the conventional drug applications. However, the fate of pbENPs developed for veterinary applications is poorly understood. pbENPs undergo biotransformation as they are transferred from one ecosystem to another, and these transformations greatly affect their impact on health and the environment. This review addresses nanoparticle fate and impact on animals, the environment, and humans from a One Health perspective.

## 1. Introduction

Antibiotic resistance, the need for increased animal production, and increased costs for medication and veterinary service continue to demand novel solutions in veterinary medicine. In response, a broad range of nanotechnology-enabled solutions has been proposed [1]. Many nanoscale delivery systems have been developed in the form of engineered nanoparticles (ENPs), a subclass of which are biodegradable ENPs (pbENPs). pbENPs are formed from polymers, which are organic macromolecules composed of repeating units organized in a chain-like architecture, and are characterized by various compositions, structures, and properties, capable of being decomposed by the living organisms [2,3]. Due to their versatility, polymeric nanoparticle systems are well suited for specific biomedical applications. Many types of pbENPs have been developed as delivery systems [2] for drugs that can be either entrapped in the polymeric matrix or conjugated to the polymer [4]. From a total of more than 8000 research papers focusing on delivering medical, veterinary, and agricultural active ingredients published between 1990 and 2020, only 3% are focused on veterinary applications, 4% on agricultural applications, and the rest of 93% on medical applications [5].

The pbENPs used in nanomedicine are categorized based on the type of polymer used: natural or synthetic molecules. According to Jarai et al. (2020) [6], important natural polymers include polysaccharides, such as chitosan, cellulose, and hyaluronic acid, as well as natural proteins. The synthetic polymers most often used in nanomedicine are: (1) Polyesters ((poly(glycolic acid) (PGA), poly(lactic acid) (PLA), poly(lactic-co-glycolic acid) (PLGA), poly(ε-caprolactone) (PCL)); (2) polyanhydrides; (3) polyamines ((Poly(ethylenimine) (PEI)); (4) Temperature-responsive polymers, such as poly(N-isopropylacrylamide) (PNIPAM); and (5) pH-responsive polymers, such as acetals, hydrazones, and diorthoesters. 

Given their therapeutic advantages, entrapment or encapsulation of various drugs in polymeric biodegradable nanoparticles has been investigated. Nanoparticles were developed to deliver hormones, antibiotics, genes, anticancer drugs, anti-inflammatory drugs, antigens, and growth factors [7,8,9,10,11,12,13]. Such systems can be used for targeted delivery of different cargo molecules [14] to improve drug bioavailability [15], to sustain drug effects in certain target tissues/organs [16], to solubilize drugs, and increase the stability of drugs against degradation [17]. pbENPs, with a mean diameter less than 150 nm, have been shown to possess advantages in delivering antibiotics, hormones, and vaccines for veterinary use [18], and have become an important innovative tool in veterinary science. For veterinary applications in general, it is important that the nanocarrier increase the circulation time of the incorporated active substance, while also ensuring renal elimination [5,19]. The most common main components used for pbENPs synthesis are summarized in Table 1.

With extensive developments in pbENPs application as veterinary drug delivery systems, the risk they pose to animals, the environment, and to humans has become a concern. As in human medicine, pbENPs encounter different biological environments en route to the site of action, as well as ex vivo after excretion. As a result of these exposures, pbENPs biotransform to materials that could detrimentally impact the health of the consumer of livestock products (i.e., the human) or the environment. In animals, as in humans, the biological barriers encountered by pbENPs in vivo are diverse, including elimination by the mononuclear phagocyte system, blood rheology and fluid dynamics of blood flow, cell membrane permeability and consequent endosomal accumulation, and removal via drug efflux pumps [20,21]. In this framework, extravasation of pbENPs is a factor that needs to be assessed and understood not only to ensure the safety of animals, but also to establish to what extent and structure pbENPs arrive in the environment, and how such (nano)materials interact with non-target species, including humans. Ultimately, pbENPs will readily transform and have altered characteristics as they are transferred between one biotic or environmental compartment to another, and these poorly understood transformations will dramatically affect their impact on health and the environment. To address critical knowledge gaps, this review examines the fate of pbENPs developed for veterinary applications, specifically at the intersection of the animal-environment-human matrix. To provide the highest level of understanding of the fate of pbENPs designed for use in veterinary practice, an integrated approach is necessary. One such approach could be based on the One Health model by taking a closer look at the interconnectivity between the three components (animals, environment, and humans) underlying the One Health perspective. A series of three case studies are discussed, with the goal of understanding the pbENPs fate needed to ensure optimal health outcomes, minimized risks, and low environmental impact of these particles when used for veterinary applications, specifically in livestock. A further aim is to provide a rich source of data that can be further used to establish an intelligent strategy for pbENPs use in veterinary medicine, as well as informing existing regulatory bodies in drafting new rules and regulations.

**Table 1:** Summary of main components used in common pbENP synthesis.

Polymers	Surfactant/Crosslinkers	Methods	Organic Phase	Final Format	References
Poly(lactic-*co*-glycolic) acid–PLGA Hydrophobic, option in monomer ratios and Molecular weight	Poly(vinyl alcohol)–PVA Tween 80 Pluronic family Myritol 318 Span 60 Span 80 Poloxamer 408–407 Polyethylene glycol-PEG	Emulsion evaporation Sonication Microfluidization Nanoprecipitation Mixing Microfluidizer	Ethyl acetate DCM DMF Acetone	Powder: Freeze dryer Spray drier	[22,23,24,25]
Poly(lactic acid)–PLA Hydrophobic, several Molecular weight					
Poly(ε-caprolactone)–PCL Hydrophobic and several molecular weights					
Lignin-graft-PLGA Amphiphilic Several MW	No surfactants	Emulsion evaporation Sonication Microfluidization	Ethyl acetate DCM DMF Acetone	Powder: Freeze dryer Spray drier	[26,27]
Chitosan Cationic and pH sensible Several MW	Sodium tripolyphosphate (STTP)	Ionic gelation	Water based	Liquid/powder	[28,29,30]
Sodium alginate Anionic Several MW	CaCl_2_ CaCO_2_	Ionic gelation Emulsion gelation	Water based	Liquid/powder	[30,31]
Zein (corn protein) Hydrophobic MW of 20–30 kDa	Tween 80 DMAB SDS	Nanoprecipitation with sonication or Microfluidization	Acetone Alcohol	Liquid/Powder	[32,33]
Carboximethyl cellulose Hydrophilic, anionic Carboximethyl chitosan Hydrophilic, cationic	Poly(vinyl alcohol)–PVA PEG Sulfuric acid 4-aminobenzaldehyde	Nanoprecipitation with sonication	Water based	Oven dry	[34,35]

Notes: DCM, dichloromethane; DMAB, didodecyldimethylammonium bromide; DMF, dimethylformamide; DMAB, didodecyldimethylammonium bromide; SDS, sodium dodecyl sulfate.

## 2. Antibiotic-Loaded pbENPs

The emergence of multidrug resistance in bacteria has become a global challenge in treating infections in both human and veterinary applications. Approximately 700,000 human deaths are due to infections caused by multidrug-resistant bacteria every year across the globe, and this figure is expected to increase to 10 million by 2050 [36,37]. According to Ezzariai et al. (2018) [38], the concentrations of several veterinary antibiotics, such as tetracyclines, fluoroquinolones, macrolides, and sulfonamides, ranged between 1 and 136,000 g kg^−1^ of dry matter in sludge and manure—which can contribute significantly to the development and spreading of resistant bacteria. Antibiotic resistance phenomenon may be caused by numerous reasons, including low bioavailability, improved action of efflux pumps to excrete drugs from the bacterial cell, and expression of resistance genes [39]. Current shortcomings in combating antibiotic resistance have stimulated research on antibiotic-loaded pbENPs, with published studies increasing rapidly [1,4,40,41,42,43]. The number of research articles focused on antibiotic nano-delivery increased from 10 papers to over 70 publications over the past eight years (The number of publications focused on antibiotic-loaded NPs; the ISI Web of Science Core Collection Clarivate Analytics was searched with the following keywords: “polymeric” “nanoparticles”, and “antibiotics”—updated to February 2020). To date, the application of pbENPs for delivering antibiotics has been investigated for treating infections by *Mycobacterium tuberculosis* [44,45,46], *Pseudomonas aeruginosa* [47,48,49,50], *Staphylococcus aureus* [12,48,51], *Escherichia coli* [12,48,52], *Brucella* spp. [53] and several multidrug resistance bacteria [54].

There are several advantages offered by pbENPs that could contribute to a decrease in antibiotic resistance as described by Parisi et al. (2017) [55]: (1) Protection of encapsulated cargo from bacterial enzymatic inactivation or resistance of antibacterial polymers from enzymatic degradation; (2) the targeting specific site of infection ensuring safe delivery of high doses; (3) the enhanced uptake or reduced efflux of the antimicrobial system; and (4) more effective management in biofilm resistance [56,57]. Moreover, from a practical point of view, controlled release of the antibiotic cargo could help decrease the number of administrations, while assuring increased and constant plasma concentrations for improved treatment efficacy [58]. While the current scientific literature is promising, much work remains to be done to fully understand the potential of and mechanisms by which bpENPs may address antibiotic resistance.

In general, antibiotic-loaded pbENPs will be considered the safest and most effective when designed for a very specific task. This is because the use of antibiotics in veterinary medicine is confounded by a large diversity of species, doses, protocols, routes of administration, and types of infection. For intelligent testing strategies that consider the risks for the administered animals, the risk to the environment and humans should also be considered.

In spite of these considerations, there have been significant efforts to develop and test antibiotic-loaded pbENPs, as noted above, without taking into account the potential impact of these delivery systems on the environment and people. As of today, the research generally shows that nano-delivery systems improve the stability, release, and bioavailability of antibiotics; however, there are several important issues that need to be addressed, such as the fate of the delivery systems, or the risk for promoting antibiotic resistance when such NPs are misused used in vivo. These questions are critical—given that veterinary applications are in a leading place, in terms of public health and biosecurity challenges posed by the antibiotic resistance phenomenon [59].

### 2.1. Case Study One: Antibiotic-Loaded pbENPs-Oral Delivery

#### 2.1.1. General Implications–Oral Delivery

‘If adaptability is the best definition of intelligence, then bacteria are much smarter than we are’ [60].

The first case study will focus on the use of pbENPs for oral delivery of antibiotics. Oral delivery of drugs in veterinary practice is preferred over parenteral injections because of its simplicity and the fact that it does not require specialized training. Oral drug delivery systems involve different mechanisms of drug release to ensure a high active substance absorption, and in general, these formulations successfully protect the active ingredient from degradation and facilitate analyte absorption through the intestinal mucosa [61]. According to Ensign, Cone, and Hanes (2012) [62] despite these potential advantages, pbENPs designed for oral delivery have several potential shortcomings, such as: (1) Poor stability in the gastric environment; (2) low solubility and/or bioavailability; and (3) poor drug penetration and subsequent absorption, due to the mucus barrier. A recent review of in vitro techniques for evaluating pbENPs for orally delivered [63] has confirmed the advantages provided by chitosan NPs for oral delivery of drugs. Regardless of the wealth of information acquired in vitro, eventual in vivo evaluation is required to validate the actual performance of an oral pbENPs delivery system [64]. In nanomedicine in general, the study of mechanisms involved in permeability and absorption improvement of compounds by oral delivery in vivo has been less investigated compared to the level of attention given to material design prior to application and to in vitro testing. Although the in vivo data is limited, the most important route of eliminating orally administered drug-loaded pbENPs is via feces [65]. Therefore, to avoid environmental contamination, pbENPs need to be designed so that the active substance is fully released and absorbed in a specific time interval, consistent with the gastrointestinal (GI) transit time [12]; otherwise, the particles will be excreted in feces. Nanoscale approaches are particularly useful here as one can engineer pbENPs accounting for variations in terms of GI time depending on species, age, condition, and diet [66] or on different pathologies that could be characterized by hyperperistalsis. Moreover, there are differences in terms of gastric and intestinal composition in different species that can be accounted for. This species-specific physiology could increase or decrease the degradation of both the pbENPs carrier and the active ingredient.

Information on the transfer of pbENPs from animals to the environment is a topic that has not been actively studied, but given the number of research papers published on antibiotic-loaded pbENPs, the issue clearly warrants a discussion. Based on the dynamics of the antimicrobial treatment in animals, it is necessary to consider possible contamination routes of the environment either with an active substance when still physically associated with the pbENPs, or by the separate pbENPs and antibiotic after release.

In the event of antibiotic excretion, selective pressure in the environment could lead to developing resistant ‘superbug’ bacteria. The implication of antibiotics administered in veterinary medicine in selecting such bacteria has already been demonstrated and is not the focus of this review [67]. However, it is important to emphasize that increased bioavailability of antibiotics incorporated in pbENPs relative to the conventional antibiotic in vivo should contribute to a decrease in the amount of analyte released into the environment.

If the antibiotic is not completely released from the carrier prior to excretion, there is an increased risk of environmental contamination not only with active substances, but also with the antibiotic-loaded pbENPs. Once in the environment, the nanoparticles will degrade over time, making the antibiotic available in small doses over a longer time interval. As the environment, animals, and humans are connected, there is a risk of contamination of humans with such ‘superbugs’ after their selection in the environment. Additionally, the link demonstrated in the literature between the antibiotics used in livestock and the development of antibiotic resistance in humans consuming animal products is of consideration here as well [68].

#### 2.1.2. Enrofloxacin-Loaded PLGA NPs

For a better understanding of how antibiotic-loaded pbENPs could reach various components of the One Health System, for case study 1, we consider the study of enrofloxacin-loaded poly(lactic-co-glycolic acid) (PLGA) nanocarriers designed for oral delivery by Paudel et al. (2019). As enrofloxacin is a veterinary antibiotic used in the therapy of several species, we chose swine as the model species. Depending on the pathology (mostly respiratory or digestive), a general treatment with antibiotics is often required for a wide range of infectious diseases characterized by high morbidity. Therefore, in the intensive farming system, oral delivery is preferred instead of parenteral delivery to reduce the stress of the animals. In swine, enrofloxacin is used to treat several bacterial infections, such as *Paseurella*, *Mycoplasma*, *E. coli*, or *Salmonella*. According to The European Agency for the Evaluation of Medicinal Products (*Committee For Veterinary Medicinal Products)*, the recommended dose for swine is 2.5 to 5 mg enrofloxacin/kg bw/day for 3 to 5 days [69]. According to the same agency, after oral administration, the bioavailability in rats was estimated to be 75%, although elimination was rapid via both urine and feces. The usual withdrawal period for enrofloxacin is 10 days after the end of the treatment.

Enrofloxacin-loaded PLGA NPs could be delivered orally in a suspension, for example, in drinking water, to ensure that environmental contamination is minimized by using automatic water dosing devices. However, contamination of the local environment is still likely because of the feeding behavior of animals in intensive farming systems; farmers, veterinarians, and professional workers could be exposed to antibiotic-loaded pbENPs in the process, as well.

The nano-enabled antibiotic designed by Paudel et al. (2019) was shown to release the therapeutic (in vitro) over five days (96%), making it suitable for the treatment. However, this longer release, combined with variable gastrointestinal transit time, could be responsible for eliminating enrofloxacin loaded PLGA NPs via feces. Depending on the manure management strategy of the farm, the NPs could reach various components of the environment, as well as humans. The extent to which the pbENPs could contaminate the environment and lead to human exposure will be dependent on NPs degradation. When degradation is complete, the active substance should have the same behavior as in conventional therapy.

Paudel et al. (2019) also showed that for *E. coli*, the minimum inhibitory concentration was reduced by ≈23% compared to free enrofloxacin alone. This, combined with increased bioavailability, could be an interesting element to reduce the dose of enrofloxacin, and therefore, its side effects—including the propagation of antibiotic resistance.

Therefore, the two most important control measures to avoid negative interactions with people and the environment are directly related to 1—pbENPs design and degradation kinetics, and 2—on-site farm management practices. First, pbENPs could be specifically engineered to release the antibiotic and to degrade according to species-specific gastrointestinal transit time. Second, in terms of management on the farm, the water feeding system should be automatic, and the manure and wastewaters should undergo longer-term treatment or storage to ensure complete degradation of the pbENPs.

As is the case for conventional therapies, before the introduction and application of antibiotic-loaded pbENPs, an investigation of antibiotic resistance phenomena as a function of low-level analyte release is strongly recommended [60]. It is clear that the assessment of the long-term effects of the use of pbENPs must occur prior to their deployment into the veterinary pharmaceuticals market. Even within this framework, one must recognize that the only hope one can have is not to prevent the emergence of resistance, which is a stochastic phenomenon, but rather to delay its spread [60].

Most protocols that involve antibiotics are based on routes of administration, the pharmacokinetics, and pharmacodynamics of the drug, as well as the dose scenario and species. Unfortunately, these protocols are not always followed, and inappropriate overuse of antibiotics in veterinary science is an important cause of antibiotic resistance [70]. Clearly, there are new safety challenges imposed by the use of pbENPs as compared with conventional drug strategies. The above case study offers a general view of the fate of orally delivered antibiotic-loaded pbENPs from a One Health perspective and highlights a number of these unique concerns.

## 3. Vaccine Loaded pbENPs

Today, the vaccination of livestock represents the most useful targeted prevention tool against infectious diseases [71]. Vaccinations involve and influence different components of the One Health umbrella, often in unanticipated ways. For example, Marsh et al. (2016) [72] showed that livestock vaccinations translate into increased human capital and school attendance by girls in sub-Saharan Africa. However, Jorge and Dellagostin (2017) [71] did identify series of disadvantages of conventional vaccines, several of which are relevant to the current review: (1) For live-attenuated vaccines: Live strains are not highly protective, reversion to virulence to a more virulent phenotype can occur, and there is a need for refrigerated storage; (2) for inactivated vaccines: They cannot provide effective long-term protection, due to the destruction of the pathogen replication processes; (3) for recombinant subunit: The need for an adjuvant; and (4) for RNA/DNA-based: Low stability. Although conventional vaccines will continue to be used and developed in the future, nano-enabled new approaches are emerging rapidly, and more importantly, could address several of the existing shortcomings of conventional vaccines. This is highlighted by the high number of publications in this field, totaling 62 research papers and reviews published in 2019 (The number of publications for vaccines that are based on pbENPs as a delivery system; ISI Web on Science Core Collection Clarivate Analytics was searched by the keywords: “polymeric”, “nanoparticles”, and “vaccine”—updated to February 2020). As pathogens continue to emerge and evolve, interest in economical vaccines promoting efficient immune responses has become significant [73]. pbENPs are ideal candidates, due to their biocompatibility, predictable and controllable degradation, and diverse and tunable chemistry [74].

For optimal response, various antigens can be incorporated into or onto nanoscale engineered carrier molecules capable of stimulating the immune system. pbENPs can offer multiple advantages over conventional antigen delivery systems: (1) Protection against degradation; (2) controlled release of the antigen; (3) modification to target certain immune cells; (4) being of similar size to viral pathogens, therefore they are efficiently taken up and internalized by antigen-presenting cells; (5) useful as a carrier for both antigens and adjuvants, ensuring co-delivery to the same destination; and (6) may limit the distribution of certain antigens, and thereby reduce the required dose and the possible side effects [75]. To date, several viral antigens [76,77], as well as some with zoonotic potential [73,78,79,80], have been incorporated into pbENPs. These studies have demonstrated that pbENPs are at least equivalent to traditional platforms in terms of efficacy, and in certain cases, superior for inducing a cell-mediated immune response. For example, Yang et al. (2020) successfully achieved the incorporation of Newcastle disease virus (NDV) in chitosan (CS), hydroxypropyl trimethyl ammonium chloride chitosan (HCS), and sulfated chitosan NPs. The results showed that the NDV-loaded CS and HCS/CS NPs induced sufficient humoral immunity levels (HI > 5) and higher cellular immunity levels than did the commercial oil emulsion vaccine. Additionally, the protective rates of these nanoparticles against highly virulent NDV reached 100%, indicating a high potential for CS and HCS NPs to be used against Newcastle disease. The use of NP delivery systems can enhance vaccine effectiveness and ensure better delivery through platforms that are specifically tailored for each species [81].

While the nasal and pulmonary route of vaccine administration is not as common for humans, it is often used in veterinary medicine. Additionally, some studies have focused on the use of pbENPs as a tool for oral vaccine administration [82], therefore creating new potential for more complex antigen delivery.

Materials that overcome delivery barriers determined from human medical research have been translated into use on livestock and poultry vaccines. The relatively easy and inexpensive manufacturing process for certain polymeric nanoparticles (i.e., PLGA-NPs)—making them attractive for the vaccine market [83].

It is expected that both research and translation of pulmonary vaccine delivery using NPs in livestock and poultry will rapidly expand [81]. Unfortunately, many of the mechanisms of vaccines based on pbENPs remain unclear, and the environmental health and safety profile of the delivery system are poorly understood. To fill these knowledge gaps, further research is necessary to assess the safety of both target and non-target species, while ensuring that animals are managed properly to guarantee the maximum vaccine efficacy.

### 3.1. Case Study 2: Vaccine Loaded pbENPS for Pulmonary Delivery

#### 3.1.1. General Implications–Pulmonary Delivery

To overcome the disadvantages of conventional drug delivery systems, a lot of research now aims at the development and use of novel delivery platforms, including those operating at the nanoscale [84]. Pharmaceutical-loaded pbENPs can be delivered by aerosolization as a liquid suspension or a dry powder that dissolves after contacting the aqueous environment of the lung epithelium [85].

As is true for other delivery routes, the physicochemical properties of pbENPs also significantly influence their fate and deposition in the lungs [86]. Understanding the fate of nanoparticles in the lung as a function of their properties is critical for developing more effective inhalable formulations [87].

Within the host, the fate of particles entering the upper respiratory system is dependent on aerodynamic properties, such as impaction, sedimentation, and diffusion, as reviewed elsewhere [81]. The upper airway system is lined with a thick mucus layer, which acts as a protective layer to trap and clear particles [88]. Mucociliary movements clear foreign particles by coughing or swallowing before they can move to the lower respiratory system [89]. The clearance in this region and possible release of pbENPs into the environment depends on elements specific for each species, such as the number of cilia and the ciliary beat frequency or the amount and composition of the mucus [90]. In terms of biological parameters described above, besides obvious differences between categories of species used for production, such as ruminants, swine, and poultry, variations within the same species also exist [91]. Various elimination pathways for nanoparticles from the lungs include dissolution, mucociliarly escalator, translocation from the airways to other sites, phagocytosis by macrophages, neuronal uptake, and coughing [92]. Therefore, even after their use, nanoparticles can be expelled, contaminating the environment and posing a risk to non-target species, including humans.

#### 3.1.2. Influenza Antigens Encapsulated in PLGA NPs

For a better understanding of how drug-loaded pbENPs could reach various components of the One Health umbrella, for case study 2, we consider the influenza vaccine designed for mucosal delivery developed by Alkie et al. (2018) [93]. As avian influenza is a major disease and an important zoonosis, we chose poultry as the model species.

Avian influenza virus (AIV) is an extraordinary complex pathogen, that can cause significant losses in domesticated poultry. The disease is prevalent worldwide and often associated with important economic losses among broilers and layers. Moreover, due to its zoonotic potential, it can threaten human life with looming pandemic potential [94]. In endemic regions, besides biosecurity, the specific immunoprophylaxis measures remain the most important means of disease prevention and control. Several approaches have been attempted so far, including the use of pbENPs for enhancing the mucosal immune response along the respiratory tract [93,95,96,97].

In the intensive farming system, nebulization in closed spaces is widely used to deliver vaccines, including the ones against avian influenza. The avian influenza antigens encapsulated in PLGA NPs by Alkie et al. (2018) [93] were shown to release the antigen (in vitro) over three weeks. Theoretically, this could be a major advantage in terms of immune stimulation, as a long release could eliminate the need for a booster vaccination. Nevertheless, as the nebulization is carried out in closed spaces, there is a risk for contamination of the environment, not related to characteristics, such as size and density, but to their long release. Although the process of nebulization can be relatively well controlled for companion animals, there are several challenges hindering the implementation of the nano-enabled delivery method in intensive farming systems, such as poultry, where nebulization is currently largely used for vaccination. Even if a precise dosage is previously calculated, some pbENPs containing the vaccine could reach the environment, and farmers, poultry workers, and veterinarians could be exposed to these pbENPs.

One example of how the NPs can influence and mislead the interpretation of results is the study of Wu et al. (2019) [98], where the authors are assessing the transmission risk of avian influenza virus among poultry supply chains in Guangdong, China. During the survey, poultry workers (*n* = 296) and general population (*n* = 232) were tested by using serology. In a scenario where an influenza vaccine that is incorporated in pbENPs, with a release of the active substance for more than three weeks contaminates the environment, individuals making contact with the delivery system could develop antibodies against the virus, therefore misleading the results, as in most cases serology cannot make the difference between immunoglobulins developed after vaccination and the ones developed after contact with the wild strain of the virus. Indeed, there are several environmental stressors, affecting the survival of influenza antigens—of which, direct sunlight seems to be the most important [99]. Such factors could influence the decay rate, but since the antigens are protected by the pbENPs, even if the process is slightly accelerated, environmental exposure is highly probable. As discussed in case-study one, the most important control measures are directly related to pbENPs design and on-site farm management. Similarly, pbENPs could be specifically engineered or tuned up to match specific requirements, such as release long enough to ensure an optimal immune response, and short enough to minimize environmental or human contamination.

There are different safety challenges imposed by the use of pbENPs based vaccines as compared with antibiotic-loaded pbENPs, mainly based on the active substance and not on the delivery system. For example, if antibiotics are used in livestock only when a certain pathology is identified, vaccines are systematically used worldwide as part of disease control strategies. Therefore, if pbENPs based vaccines will be implemented on a large scale, their impact on the environment, animals, and people will be directly proportional with their use and the type of antigen used.

Besides the poultry industry, aerosol administration may be practical in the swine industry, due to the close proximity and smaller housing facilities [81]. It has been shown that an aerosolized vaccine against influenza in swine was able to either protect the animals or reduce the virus load when challenged with the specific antigen [100,101]. Morgan et al. (2016) [100] suggest that in swine, local lung immunity plays an important role in the protection against influenza. Additionally, Lee et al. (2018) [102] developed a polymer-templated protein nanoball with direction-oriented hemagglutinin1 on the surface (H1-NB) as a new influenza vaccine on mice. The authors showed that H1-NB efficiently stimulated H1-specific immune activation, which subsequently prevented H1N1 virus infection in mice. Theoretically, a vaccine like this could be delivered via pbENPs, and the NPs properties should enhance the local immune response. Nevertheless, the zoonotic potential of such a virus could make it undesirable without an extensive safety profile.

## 4. Hormone Loaded pbENPS

Nano-enabled delivery of hormones has emerged as a new pharmacological approach for the diagnosis and treatment of reproductive problems, for oestrus detection and sperm freezing, and for direct calving interference. pbENPs have been proposed as effective platforms for the protection and controlled release of reproductive hormones, including steroid or gonadotropic hormones [103], as well as other applications [104,105]. A significant advantage is the ability of pbENPs to protect the hormone from degradation by seminal plasma enzymes [104,106]. Although the number of studies performed on the use of hormone-loaded NPs for veterinary purposes is quite limited (less than 10 in 2019 according to the number of publications for hormone loaded pbENPs as a delivery system; ISI Web of Science Core Collection Clarivate Analytics was searched with the keywords: “polymeric nanoparticles”, and “vaccine”—updated to February 2020), several patents have been registered [107], and a number of researchers have recommended further studies to fully evaluate the reproductive performance of several species after administration of hormone-loaded pbENPs [104]. For example, Fernández-Serrano, Casares-Crespo, and Viudes-de-Castro (2017) [104] successfully synthesized chitosan–dextran sulfate (DS) nanoparticles containing a GnRH (gonadorelin) analog, with an entrapment efficiency of 43.11 ± 6.13%. Similarly, Pamungkas et al. (2016) [108] successfully synthesized human chorionic gonadotropin (hCG) loaded chitosan NPs with a particle size of 226.2 ± 26.3 nm with entrapment efficiency of 54.1 ± 12.9%; the authors showed that the new NPs increased induction of ovulation in dairy cattle. In a study conducted on goats, Hashem and Sallam (2020) [106] showed that GnRH loaded chitosan NPs reduced the dose of GnRH by 75%, with the same positive effects on reproductive performance when compared with the classical GnRH therapy.

Given the importance of classical hormone therapy, the fate of hormones used in the beef and dairy industries, as well as the potential for human exposure, has been extensively reviewed [109]. Residues of hormones often persist in animal products consumed by humans, which may cause several health concerns, including allergic response [110]. Properties of hormone loaded pbENPs, such as controlled release and the complexity of their interaction with biological systems, raise important questions related to the use of such pbENPs relative to the One Health concept. To date, no systems for controlling or monitoring their use have been proposed. It is worth noting that the studies published so far are largely focused on the design and characterization of hormone loaded pbENPs, and there has been little focus on the fate and impact of the particles outside the host; such understanding is essential to ensure the sustainable use of hormone loaded pbENPS in livestock.

### Case Study 3: Hormone Loaded pbENPs for Parenteral Delivery

Some veterinary therapeutics, including hormones, may exert toxicity because of uncontrolled systemic distribution or non-target effects, and they require localized delivery through nano-enabled approaches [111]. Depending on the active substance and side effects, intravenous, intramuscular, or subcutaneous administration could represent safe routes of delivery for pbENPs, while also minimizing the risk for human and environmental exposure to the drug or particles themselves.

Therefore, the third case study will focus on the use of pbENPs for the parenteral delivery of hormones. We consider the study of chitosan nanoparticles of hCG (Human Chorionic Gonadotrophin) hormone in increasing induction of dairy cattle ovulation as developed by Pamungkas et al. (2016) [108]. As hormone therapy in veterinary medicine is used primarily in livestock, we chose cattle as the model species.

The NPs designed and characterized by Pamungkas et al. (2016) [108] were tested in vivo, delivered by nasal spray, and compared by intramuscular (i.m.) administration of hCG. The results showed that CS NPs with hCG can be used in increasing the induction of ovulation in dairy cattle. Unfortunately, the characterization of NPs did not include a release study. This limitation is making a risk assessment from a One Health point of view very difficult. The impact of the nanoparticles on the environment and people will depend on the release rate of the hormone, similar to the situations previously covered in case studies one and two. If the hormone loaded NPs are delivered via i.m. injection, the risk of direct environmental contamination with pbENPs is minimal, but dependent on the release time of the hormone and the route of administration. While biodistribution of drug-loaded pbENPs falls outside the scope of this review, it is important to understand how the delivery system could potentially reach the environment after administration. Wyss et al. (2020) [112] demonstrated that polymeric NPs (120 nm and high negative surface charge) functionalized with dermatan sulfate, chondroitin sulfate, heparin sulfate, and hyaluronic acid undergo fast renal clearance (74% of injected dose in the first 2 h) after intravenous administration in mice. Intact polymeric NPs were shown to be eliminated via urine. This route of elimination could, therefore, result in environmental contamination. After their elimination via urine, the fate of parenteral administered pbENPs is similar to that for oral delivery. As for the other delivery routes described previously—given the complexity of different species anatomy or renal pathologies, an assessment of risk should be done for each nanoparticle type and target species.

An example of potential direct transmission of pbENPs to humans is the misuse of hormone-loaded pbENPs in species intended for human consumption. Often, non-veterinarians are responsible for interventions and treatments of livestock [113].

In this framework, pbENPs exposure to humans and the environment could directly occur. Another possible scenario is related to a slow degradation of the polymeric matrix, yielding pro-longer release of the active substance. Given this scenario, regulations may need to be use-specific to ensure that the risk of milk or meat contamination by the active substance residues or drug-loaded pbENPs is minimized. Moreover, based on the fact that dairy products are often placed at low temperatures, a second phase of polymer degradation could occur in the consumer body and may need to be evaluated.

As for the previous two case studies, the most important control measures are directly related to pbENPs design and on-site farm management practices. First, pbENPs could be specifically engineered or tuned up so that the release of the active substance is long enough to ensure optimal effect and fast enough to avoid pbENPs or active substance remanence in meat or milk products. Second, on-site farm management should develop and apply practices to reduce the impact of contaminated urine on the environment and people.

Parenteral administration of drug-loaded pbENPs in mastitic cows, sheep, or goats [114] could increase the risk for consumers through milk consumption as compared to non-nano delivery methods, where residue elimination time is clearly established. Other examples include a product that was approved for livestock use by UE’s Committee for Medicinal Products for Veterinary Use (CVMP) in 2015 and by the US Food and Drug Administration (FDA) in 2016. The product contains as active ingredient PEGylated granulocyte stimulating factor (pegbovigrastim) in a 30 kDa protein + PEG nanocarrier to be used as prophylactic treatment against mastitis in dairy cows via subcutaneous injection.

All these factors must be taken into account by regulatory agencies in drafting new rules and regulations specific to each application with its own delivery route, type of drug, nanoparticle design, and animal species characteristics.

## 5. Regulatory Framework and Challenges

Before being placed on the market, veterinary drugs need to go through an authorization system based on the quality, safety, and efficacy of the drug. Assessments are managed by large agencies, such as the FDA in the USA, the European Medicines Agency in the European Union, or the Australian Pesticides and Veterinary Medicines Authority (APVMA) in Australia. The primary focus of such evaluation is to ensure an improvement in human and animal health. Environmental risks are also assessed and can be factored in the overall benefit/risk assessment. In the European Union, environmental risks can eventually lead to refusal of authorization (which is not the case for human drugs) [115].

Over the last decade, regulatory approaches to assess products derived from nanotechnology have been intensely discussed, and a range of measures have been implemented [116]. Common challenges across international regulatory bodies include terminology, definitions, testing methods and standards, standardized measurement, calibration, and reference materials [117,118]. Most regulatory approaches use the existing framework developed for non-nanoscale chemicals, in conjunction with a case-by-case approach [117,118]. Frameworks may evolve to incorporate grouping and read-across approaches, which are more efficient compared to case-by-case approaches. However, science is at present not advanced enough to fully substantiate decision criteria needed in risk assessment [119].

When dealing with the types of carrier systems described in this review, one question is whether there would be all considered “nano” by regulators. This can become a very complex and contentious question, as illustrated by the different definitions used within the European regulatory context [120]. Another important consideration is whether a nano-enabled veterinary drug would be considered as a new substance or as a reformulation of an already authorized substance. Addressing these questions through an iterative problem formulation process (e.g., Walker et al. (2018) [121]) will determine the need for additional data on safety or effectiveness, as applicable.

Placing a new drug on the market typically takes between 10–14 years [43], and considerable resources may be required to generate additional data specific to nano specific properties. Regulatory hurdles may, thus, slow down innovation and the improvement of currently used veterinary drugs. The new EU veterinary regulation (coming into force in 2022) prompted new discussions, and recent reports advocate the establishment of a One Health approach to evaluate veterinary drugs [115]. The implementation of the One Health approach for regulatory assessment is currently challenging, due to the gaps in our scientific knowledge—particularly around the biological behavior of nanomaterials in organisms and the environment.

## 6. Knowledge Gained and Remaining Research Gaps

There are numerous barriers that protect an organism from xenobiotic agents. In mammals, these barriers include the immune system (both cell-mediated and humoral), skin and mucous membranes, as well as the blood-brain barrier. It is possible that pbENPs could bypass some of these barriers—either due to their small particle size or to their molecular amenability to surface functionalization that could be used to incorporate desired characteristics for effective uptake and distribution [122]. Alexis et al. (2008) [123] showed that the main parameters influencing the biodistribution and elimination of NPs can be divided into two main categories: (1) Host-related features, such as physiology, including physiological deficits; and (2) nanoparticle properties, such as composition, size, core properties, surface functionality, and charge. In addition to these two main categories, we identified some specific issues of interest from the three case studies discussed. For antibiotic-loaded pbENPs, the kinetics of the antibiotic release and nanoparticle degradation need to match the GI transit time for the specific species of interest to minimize environmental impact and subsequent antibiotic resistance promotion. Excretion through feces is an important pathway to the environment for drugs that are delivered orally, and farm management protocols need to be in place to avoid environmental contamination and human exposure. For vaccine-loaded pbENNPs, application by nebulization may lead to air contamination if appropriate containment protocols are not in place. For hormone-loaded pbENPs, nanoparticles can protect the hormone analyte from degradation, which is also a benefit provided by polymeric nano-delivery systems used for other drugs. Intramuscularly administered particles can be excreted in urine, and this must be contained to avoid human exposure to the pbENPs when consuming animal products. It is evident that the fate, transformation, and elimination of pbENPs is not only a function of nanoparticle characteristics and the physiology of the host, but is also highly dependent on the administration route. Typically, the administration route is established during the development of a new drug. In animals, as in humans, the transformation of pbENPs is driven by different factors depending on the administration pathway. Thus, careful design of the engineered particles for the specific route of administration is needed for improved nanoparticle bioavailability in the host and enhanced efficacy of the delivered drug. Such design must consider in vivo transformations that may alter drug activity and distribution, and must also minimize excretion into the environment and negative consequences on the host, consumer, and the environment.

**Figure 1:**
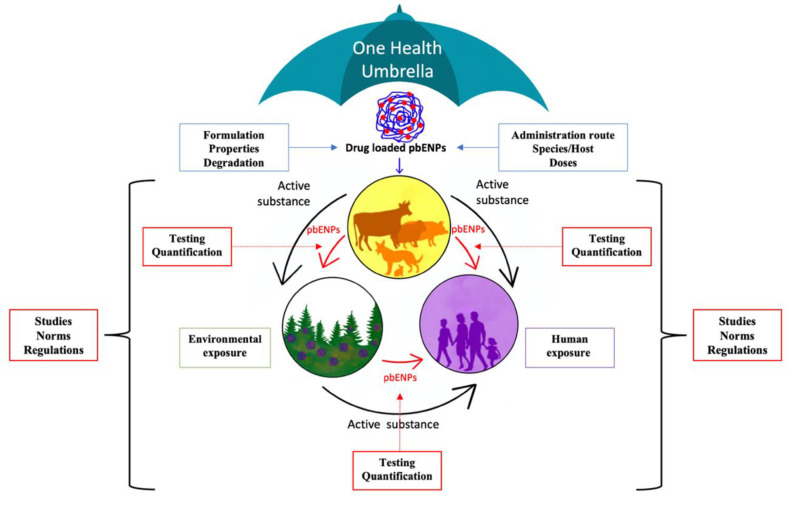
Fate of pbENPs when used in veterinary science—a One Health approach (overview).

In light of the fate of drug-loaded pbENPS and their impact on the environment and humans from a One Health perspective (Figure 1), several research gaps and challenges were identified and summarized in Table 2.

## 7. Conclusions

Remarkable progress has been made supporting pbENPs application in veterinary medicine for safe, healthy, and sustainable increases in livestock productivity. However, with these rapid advances, several challenges have emerged surrounding potential risks of pbENPs nano-delivery systems to the animals, humans, and the environment. Host-related features and nanoparticles properties are the two determinants of complex interactions that will eventually lead to human and environmental exposure. While the One Health approach could be considered efficient for summarizing the knowledge as well as the knowledge gaps, establishing a dynamic link between scientists, practitioners and policymakers will be critical to the safe use of nanotechnology in veterinary medicine. Therefore, new frameworks for global risk assessment, which go beyond the focus on generally-applicable solutions, should be encouraged.

## Figures and Tables

**Table 2:** Overview of identified challenges posed by pbENPs, as well as the resolutions from a One Health perspective.

Present Challenges	Resolutions	References
**Transformations and Contamination Pathways and Monitoring Strategies**		
**Nanoparticle properties and host-related features influencing the biodistribution of pbENPs;** **A small number of in vivo studies;**	Establish which pbENPs transformation processes are taking place and to what extent	[21,123,124]
**Host-related features influencing the elimination of pbENPs;**	Establish pbENPs routes of elimination and to what extent (e.g., the influence of the particularities of the immune system or digestive system in different species, pathologies affecting the gastrointestinal (GI) transit time, etc.)	[123,125,126]
**Nanoparticle properties and host-related features influencing the elimination of pbENPs;**	Develop and implement methods of control and detection of pbENPs eliminated by the host	[126,127,128]
**Environmental and human contamination with pbENPs during the administration;**	Develop practical and effective devices for pbENPs administration	-
**Environmental and Human Contamination**		
**Absence of guidelines;**	Establish guidelines regulations for the use of drug-loaded pbENPs in livestock depending on species, raising system, or other factors that could contribute to the exposure of humans and the environment Establish potential risks and develop corrective measures in case of environmental and human contamination	[129]
**Absence of long-time studies;**	Identify long term consequences of exposure for animals, environment, and people; encourage “green nanotechnology”	[130,131,132]
**Regulatory Issues**		
**Absence of specific regulations;**	Adapt current regulations	[115]
	Encourage fundamental research prior to development work for commercialization and build trust in nanotechnology	[129,133]
